# Mechanisms by which Factor H protects *Trypanosoma cruzi* from the alternative pathway of complement

**DOI:** 10.3389/fimmu.2024.1152000

**Published:** 2024-02-01

**Authors:** Smrithi S. Menon, Galia Ramirez-Toloza, Keith L. Wycoff, Sean Ehinger, Jutamas Shaughnessy, Sanjay Ram, Viviana P. Ferreira

**Affiliations:** ^1^ Department of Medical Microbiology and Immunology, University of Toledo College of Medicine and Life Sciences, Toledo, OH, United States; ^2^ Laboratory of Parasitology, Department of Animal Preventive Medicine, Faculty of Veterinary Medicine and Livestock Sciences, University of Chile, Santiago, Chile; ^3^ Planet Biotechnology, Inc., Hayward, CA, United States; ^4^ Division of Infectious Diseases and Immunology, University of Massachusetts Medical School, Worcester, MA, United States

**Keywords:** alternative pathway, Factor H, Factor H-related protein-5, *Trypanosoma cruzi*, evasion strategy

## Abstract

Chagas disease, a chronic disabling disease caused by the protozoan *Trypanosoma cruzi*, has no standardized treatment or preventative vaccine. The infective trypomastigote form of *T. cruzi* is highly resistant to killing by the complement immune system. Factor H (FH), a negative regulator of the alternative pathway (AP) of complement on cell surfaces and in blood, contains 20 short consensus repeat domains. The four N-terminal domains of FH inactivate the AP, while the other domains interact with C3b/d and glycan markers on cell surfaces. Various pathogens bind FH to inactivate the AP. *T. cruzi* uses its trans-sialidase enzyme to transfer host sialic acids to its own surface, which could be one of the approaches it uses to bind FH. Previous studies have shown that FH binds to complement-opsonized *T. cruzi* and parasite desialylation increases complement-mediated lysis of trypomastigotes. However, the molecular basis of FH binding to *T. cruzi* remain unknown. Only trypomastigotes, but not epimastigotes (non-infective, complement susceptible) bound FH directly, independent of C3 deposition, in a dose-dependent manner. Domain mapping experiments using 3-5 FH domain fragments showed that domains 5-8 competitively inhibited FH binding to the trypomastigotes by ~35% but did not decrease survival in complement. FH-Fc or mutant FH-Fc fusion proteins (3-11 contiguous FH domains fused to the IgG Fc) also did not kill trypomastigotes. FH-related protein-5, whose domains bear significant sequence identity to all known polyanion-binding FH domains (6-7, 10-14, 19-20), fully inhibited FH binding to trypomastigotes and reduced trypomastigote survival to < 24% in the presence of serum. In conclusion, we have elucidated the role of FH in complement resistance of trypomastigotes.

## 1 Introduction

Chagas disease (CD) or American trypanosomiasis is a chronic disabling parasitic disease caused by the protozoan *Trypanosoma cruzi* (*T. cruzi*), which is transmitted to humans and animals by a triatomine insect vector (reduviid bug). Worldwide, six to eight million people are affected, with an estimated 300,000 CD cases reported in the US (1–3). Although the disease is endemic in regions of Mexico, Central America and South America, it is slowly spreading to other regions, including the US (4). The overall global economic impact of CD is ~ $7.19 billion per year with 10% of the cost being shouldered by the US and Canada (5). Acute infections are often not diagnosed and after an asymptomatic phase that lasts 10-20 years, ~30-40% of infected people progress to the chronic stage where they develop a mega syndrome (enlargement of the heart, esophagus, and colon), resulting in death (4). Currently there are no vaccines available for *T. cruzi* infection, and no standardized treatment available for chronic Chagasic patients (4).

*T. cruzi* is an obligate intracellular parasite that can infect and cause disease in humans and various species of mammals (6). Parasite transmission to the host occurs when the triatomine insect vector is obtaining a blood meal, releasing infective trypomastigotes in the feces as it feeds. The trypomastigotes enter the host via the bite wound or via penetrating mucosal or conjunctival surfaces (7). There are also non-vectorial routes of transmission such as during transfusion, transplantation and congenitally. Once inside its mammalian host, the trypomastigotes will enter the cells and transform into infective amastigote forms and then eventually revert back to infective trypomastigotes. These processes lead to the rupture of the cells, releasing trypomastigotes into the blood stream that infect other cells or are engulfed by the vector while feeding. In the vector, the trypomastigotes revert to non-infective epimastigotes in the anterior mid gut, multiply, transform into trypomastigotes in the hind gut, and are released in feces or urine during the vector’s blood meals, thus perpetuating the parasite life cycle.

*T. cruzi* has adopted a diverse range of survival strategies that cripple the host’s innate and adaptive immune responses (8). In this context, the evasion of the complement system, a critical arm of the innate immune system, plays a central role in the development of both acute and chronic stages of the disease (4). The complement system can be activated spontaneously or in response to danger signals, such as invading pathogens, resulting in direct killing of the pathogens and can bridge innate and adaptive immunity by recruiting immune cells to sites of invasion, initiating phagocytosis, and enhancing cellular immune responses (9). The complement system is activated by three main pathways: classical (CP), lectin (LP) and alternative (AP). All 3 pathways converge to a central complement component, C3 (9). Both CP and LP have recognition molecules that recognize and bind to pathogen or cell-bound immunoglobulins or carbohydrate molecular patterns on foreign surfaces, respectively (10, 11). In contrast, AP is in a state of very low-grade activation as a result of spontaneous hydrolysis of C3 to form C3(H_2_O), a process termed ‘C3 tick-over’ (12). C3(H_2_O) binds complement protein Factor B (FB) and complement protein Factor D (FD) cleaves FB to Bb and Ba, resulting in the formation of a fluid-phase enzymatic convertase C3(H_2_O)Bb. This convertase can then cleave free C3 into C3a and C3b that covalently binds nearby surfaces. Surface-bound C3b recruits complement proteins FB and FD to form a surface-bound C3 convertase, C3bBb, which continues the complement activation process. While the contribution of the C3 tick over mechanism to initiate the AP is debated, the role of the AP in amplifying C3b deposited on surfaces through the CP and LP pathways is well-established (13, 14).

To avoid and protect the host from unwarranted damage, regulation of the complement system is essential and is carried out by various host regulators. Although complement regulation is crucial for the host, various pathogens including bacteria (15, 16), fungi (17), parasites (18) and viruses (19) adopt survival strategies that take advantage of the host complement regulators to prevent complement-mediated attack (20). Hijacking Factor H (FH), a host negative regulator of the AP, is one such strategy (21). FH is a complement protein found in blood at a wide concentration range between 116-810 μg/ml (22–24) and consists of 20 homologous domains that acts as a negative regulator of the AP in both the fluid phase of blood and on cell surfaces. FH regulates the AP on cell surfaces by recognizing select host cell glycan markers such as polyanions (e.g., sialic acids and glycosaminoglycans) in combination with deposited complement C3b fragments (that result from complement activation). Once bound, FH can inhibit complement activation by: (i) causing decay acceleration of the enzymatic complexes formed during complement activation (25–27); (ii) acting as a cofactor for another complement protein, Factor I, that cleaves active C3b into iC3b, which cannot form new enzymatic convertases (27–29); (iii) competing with FB for C3b binding (30). The N-terminus of FH (domains 1-4) carries out the complement regulatory functions while the rest of the molecule has various domains that bind to C3b and polyanions (domains 6-8, 11-15 and 19-20) found on the cell surface (31). The C-terminal domains, 19-20, are also defined as the most important domains for binding to cell surfaces. Domain 20 is the only known domain of FH that binds to host sialic acids (32, 33).

Pathogens have developed FH evasion strategies by modifying their surfaces to mimic host surfaces via expression of host cell markers that can bind FH or by expressing FH binding receptors to recruit and bind FH (21). *Neisseria gonorrhoeae*, *Candida albicans*, and *Trypanosoma brucei* are some examples of pathogens that recruit FH to their surface to escape complement-mediated attack (21). *T. cruzi* infective trypomastigotes and non-infective epimastigotes (normally susceptible to complement-mediated killing) can also bind FH following deposition of C3b fragments on the parasite (34, 35). *T. cruzi* has abundant surface sialic acids that are formed as a consequence of the parasite using a trans-sialidase enzyme to hijack host α2, 3-linked sialic acid to acceptor sites on the parasite surface (36, 37). The surface sialic acid is hypothesized to partially contribute to the high resistance of trypomastigotes to complement-mediated killing (38). Because of these abundant surface sialic acids present on *T. cruzi* and considering FH can potentially bind to these sialic acids using domain 20, it is hypothesized that FH may be used as an evasion strategy by *T. cruzi*. However, this has not been proven experimentally. Specifically, studies have not investigated: (i) whether FH binds directly to the parasite (in the absence of complement activation); (ii) the domains that FH uses to interact with *T. cruzi*; (iii) if or to what level FH protects trypomastigotes from complement.

In this study, we elucidate the interactions between FH and *T. cruzi*. We show for the first time that FH can bind directly to trypomastigotes (i.e., in the absence of complement activation on their surface). Competitive FH binding and parasite survival assays revealed the potential involvement of multiple FH domains in the FH interaction with the parasite and showed the critical contribution of host FH to the protection of this parasite from AP-mediated killing.

## 2 Materials and methods

### 2.1 Buffers

The following buffers were used: 0.25 M MgEGTA (0.25 M MgCl_2_ and 0.25 M EGTA [ethylene glycol tetraacetic acid], pH 7.4), 0.5 M EDTA (0.25 M EDTA [Ethylenediaminetetraacetic acid] disodium salt-2H_2_O and 0.25 M EDTA tetrasodium salt-2H_2_O, pH 7.4), phosphate buffered saline (PBS; 10 mM NaH_2_PO_4_, 145 mM NaCl, pH 7.4), 0.2% Bovine Serum Albumin (BSA, Sigma) + Hanks’ balanced salt solution with calcium^2+^ and magnesium^2+^ (HBSS^++^; Gibco), 0.2% BSA+ HBSS^++^ + 10mM EDTA.

### 2.2 Serum and complement proteins

Normal human serum (NHS) was purchased from Innovative Research. FH was purified as previously described in (28) and Factor H-related proteins (FHRs) were purchased from R&D Systems and Bon Opus Biosciences. Recombinant FH fragments (rH) were generated and purified using *Pichia pastoris* expression system as described previously (39, 40). FH fragment rH 6,7/18-20, is expressed and purified as described previously (41). Expression and purification of fusion proteins comprising human FH domains 18-20 or 6-7 fused to the Fc fragment of human IgG1(Fc1) or human IgG3 (Fc3) or FH domains 5-8 fused to the Fc fragment of murine IgG2b has been described previously (42, 43). A recombinant Factor H 6-8/10-14/18-20 with a D1119G mutation in domain 19 (rH 6-8/10-14/mut18-20), fused to human IgG1Fc, was expressed and purified using the methodology described for fusion proteins with individual FH domains (43).

### 2.3 Parasites and cells

*T. cruzi* trypomastigotes Tulahuen strain (clone C4; +lacz) (Cat # NR-18959) and Peru strain (clone C7; +lacz) (Cat # NR-18960) were obtained from BEI resources. Tulahuen strain was chosen because it belongs to TcVI DTU genotype, which is one of the most prevalent strains associated with chronic Chagas disease in the southern countries of South America and is widely cited in the literature (44). Peru strain was chosen as it belongs to TcI DTU genotype, which is found in countries of South America, isolated from chronic Chagasic patients and immunocompromised individuals with reactivation of Chagas disease (44). Both the strains are maintained as instructed by the company. Briefly, trypomastigotes were maintained by *in vitro* passaging in 100% confluent monolayers of mouse embryonic fibroblasts [BALB/3T3 clone A31 (ATCC, Cat# CCL-163)] in ATCC-formulated Dulbecco’s Modified Eagle’s Medium (ATCC) with 10% heat-inactivated fetal bovine serum (FBS) at 37°C, 5% CO_2_. The Brazil strain (+luc) (Cat # NR-46429) was used as a representative for *T. cruzi* epimastigotes, due to Tulahuen and Peru epimastigotes not being available in the BEI resources catalog at the time of this study. The epimastigotes were maintained in Liver infusion Tryptose medium [9g of liver infusion broth (BD), 5g of tryptose (BD), 1g of NaCl (Fisher), 8g of Na_2_HPO_4_ (Fisher), 0.4g of KCl (Fisher), 1g of Glucose (Thermo Fisher Scientific), 10 mg of Hemin (Sigma), and distilled water] as instructed by the company. Parasites were quantified in a Neubauer chamber by light microscopy using flagellar and parasite motility as indicator of live parasites.

### 2.4 Antibodies

The following antibodies were used: Murine monoclonal antibody to human Factor H antibody #2 (Quidel), Murine monoclonal IgG1k isotype control (Ebiosciences), Goat anti-mouse Alexa flour 488 IgG (Invitrogen) and F(ab)2 polyclonal goat anti-C3b IgG (LifeSpan BioSciences).

### 2.5 Factor H binding studies

3x10^6^ parasites (trypomastigotes or epimastigotes; as indicated in figure legends) were incubated with 0.2% BSA/HBSS^++^, FH fragments, FHRs or F(ab)2 polyclonal goat anti-C3b IgG (nM or μM; as indicated in figures) for 30 minutes followed by treatment with FH (nM or μM) or 5% NHS or 5% FH-dpl serum (as indicated in figure legends) in a total 50 μl volume. For experiments using 5% NHS or 5% FH-dpl serum, the complement reaction is stopped by adding 200 μl of 0.2% BSA/HBSS^++^ containing 10 mM EDTA. For direct FH-binding experiments with trypomastigotes and epimastigotes, 3x10^6^ parasites were incubated directly with varying concentrations (μg/ml) of FH for 30 minutes in a total 50 μl volume of 0.2% BSA/HBSS^++^. The samples were washed 2X to remove unbound FH with 200 μl of 0.2% BSA/HBSS^++^ and centrifuging samples at 4000 x g for 3 minutes. The remaining bound FH was detected using 10 μg/ml of murine monoclonal anti-human FH antibody followed by washing with 200 μl of 0.2% BSA/HBSS^++^ and centrifuging samples at 4000 x g for 3 minutes. A murine monoclonal IgG1k isotype antibody was used as a negative control for detection of FH binding. 10 μg/ml of goat anti-mouse Alexa fluor 488 IgG was added to detect bound murine monoclonal anti-human FH antibody followed by washing as indicated in previous step. 200 μl of 1% paraformaldehyde was then added to kill and fix the parasites. The samples were washed as indicated in the previous step and resuspended in 350 μl of 0.2% BSA/HBSS*^++^*, and the samples were run on a FACS Canto (BD biosciences) flow cytometer. 10,000 events were acquired from a gate encompassing the trypomastigote populations. Using FlowJo software version 10.7 (Tree Star), gating was done based on parasites positive for FH binding fluorescence, and geometric mean fluorescent intensities (GMFIs) on gated parasites were determined.

### 2.6 Survival assay to measure viability of *Trypanosoma cruzi* parasite

5x10^5^ parasites (trypomastigotes or epimastigotes; as indicated in figure legends) were incubated with PBS, FH fragments, FHRs or FH fusion proteins (nM or μM; as indicated in figure legends) for 30-45 minutes (as indicated in the figure legends) followed by treatment with 10-60% NHS (as indicated in the figure legends) for 60 minutes in a total of 50-100 μl volume. For experiments using individual fragments (rH 5-7, rH 6-8, rH 19-20), the parasites were incubated directly with fragments and NHS for 60 minutes in a total of 50-100 μl volume before stopping complement activity. NHS was added under conditions where only the AP is active (by adding 5mM MgEGTA to the reaction) or when all 3 complement pathways are active (NHS only) or under inactive complement conditions (by adding 10mM EDTA to the reaction) as required. 400 μl of cold media was added to stop complement activity. Parasite survivors were then quantified in a Neubauer chamber by light microscopy, counting flagellar and parasite motility and expressed as percentage of survival to complement.

### 2.7 Statistical analyses

Data was analyzed by unpaired t-test, one-way ANOVA with Tukey’s post-test using GraphPad Prism version 9.2.0 for Mac OS X (GraphPad Software, San Diego, California, USA), with specific method being indicated in figure legends or results section. P values less than 0.05 were considered significant.

## 3 Results

### 3.1 Epimastigotes bind Factor H only in the presence of C3 fragment deposition while trypomastigotes bind Factor H directly, independent from complement activation

The infective and non-infective forms of *T. cruzi* have varying levels of susceptibility to the AP. Epimastigotes (non-infective) forms are susceptible to the AP-mediated killing while infective trypomastigotes are resistant (45, 46). The specific susceptibility/resistance to the complement system of the epimastigote and trypomastigote strains used in this study was confirmed in survival assays. Brazil strain epimastigotes were treated with varying percentages of NHS under (i) AP-only conditions; (ii) conditions when all the 3 complement pathways are active; (iii) and inactive complement conditions (described in the Materials and Methods section 2.6). Under AP conditions, epimastigotes showed dose-dependent decrease in survival, reaching an LD_50_ at 11% NHS and less than 5% survival when all 3 complement pathways were active (**Supplementary Figure 1**). The epimastigotes showed no decrease in survival when complement was inactivated with 10 mM EDTA. When the Tulahuen (**Supplementary Figure 2A**) and Peru strain (**Supplementary Figure 2B**) trypomastigotes were treated with up to 60% NHS under the same experimental conditions, the survival was above 80% under all conditions tested.

**Figure 1:**
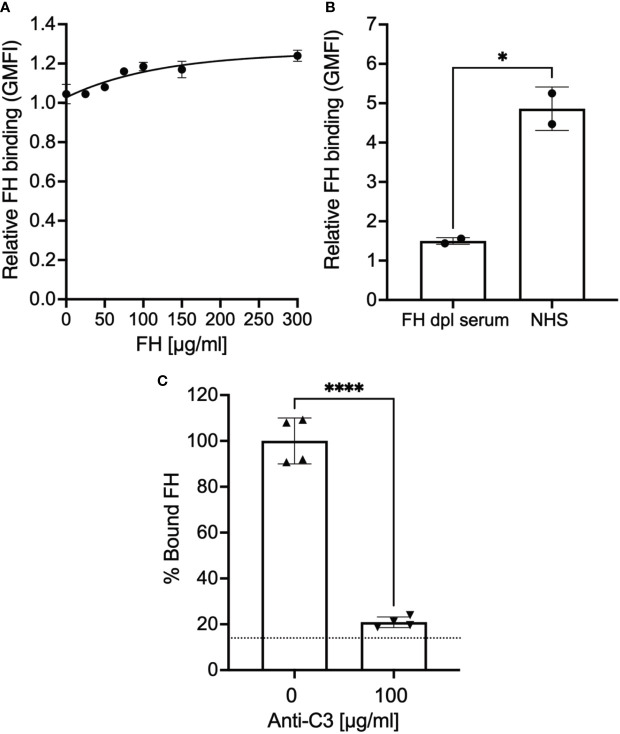
Factor H binds to epimastigotes in a C3-dependent manner. 3x10^6^ epimastigotes were incubated with 0.2% BSA/HBSS^++^ with **(A)** varying concentration of Factor H (FH), **(B)** 5% NHS or 5% FH-dpl serum, or **(C)** 5% NHS with or without polyclonal anti-C3 Ab for 30 minutes at 37°C. The complement reaction is stopped by adding 200 μl of 0.2% BSA/HBSS^++^ containing 10 mM EDTA for **(B)** and **(C)**. FH binding was determined by FACs as described in section 2.5 and was plotted relative to background for **(A)** and **(B)**. For **(C)**, FH binding was plotted as % bound FH relative to 0 μg/ml anti-C3 (100%). Results shown for **(A)** and **(B)** were representative of 2 independent experiments and was graphed as mean and standard deviation of duplicates. For **(C)**, results are shown as mean and standard deviation from two independent experiments with duplicates. Significant differences in FH binding were assessed by unpaired t test; p<0.0001****, p<0.05*. For **(C)** there were no significant differences between 100 μg/ml of anti-C3 Ab and background (represented by the dotted line).

Previous studies show that epimastigotes can bind FH in the presence of complement protein opsonization, which represents FH bound to deposited C3 fragments (34, 35). We were unable to detect pure FH protein binding to epimastigotes, in the absence of complement activation (**Figure 1A**). However, as expected, FH binding was observed when epimastigotes were incubated with 5% NHS (**Figure 1B**) and as shown in **Supplementary Figure 1A**, the parasite survival was <5% at this NHS concentration. To determine whether FH binding to the mostly dead epimastigotes is specifically due to the presence of C3 fragments from complement activation, FH binding was assessed in the presence of 5% NHS with or without polyclonal anti-C3 antibody (Ab). FH binding was reduced to baseline in the presence of 100 µg/ml of polyclonal anti-C3 Ab as compared to conditions without the Ab (**Figure 1C**). Overall, the data shows that the FH does not bind to epimastigotes unless complement activation deposits C3 fragments on the parasite surface.

**Figure 2:**
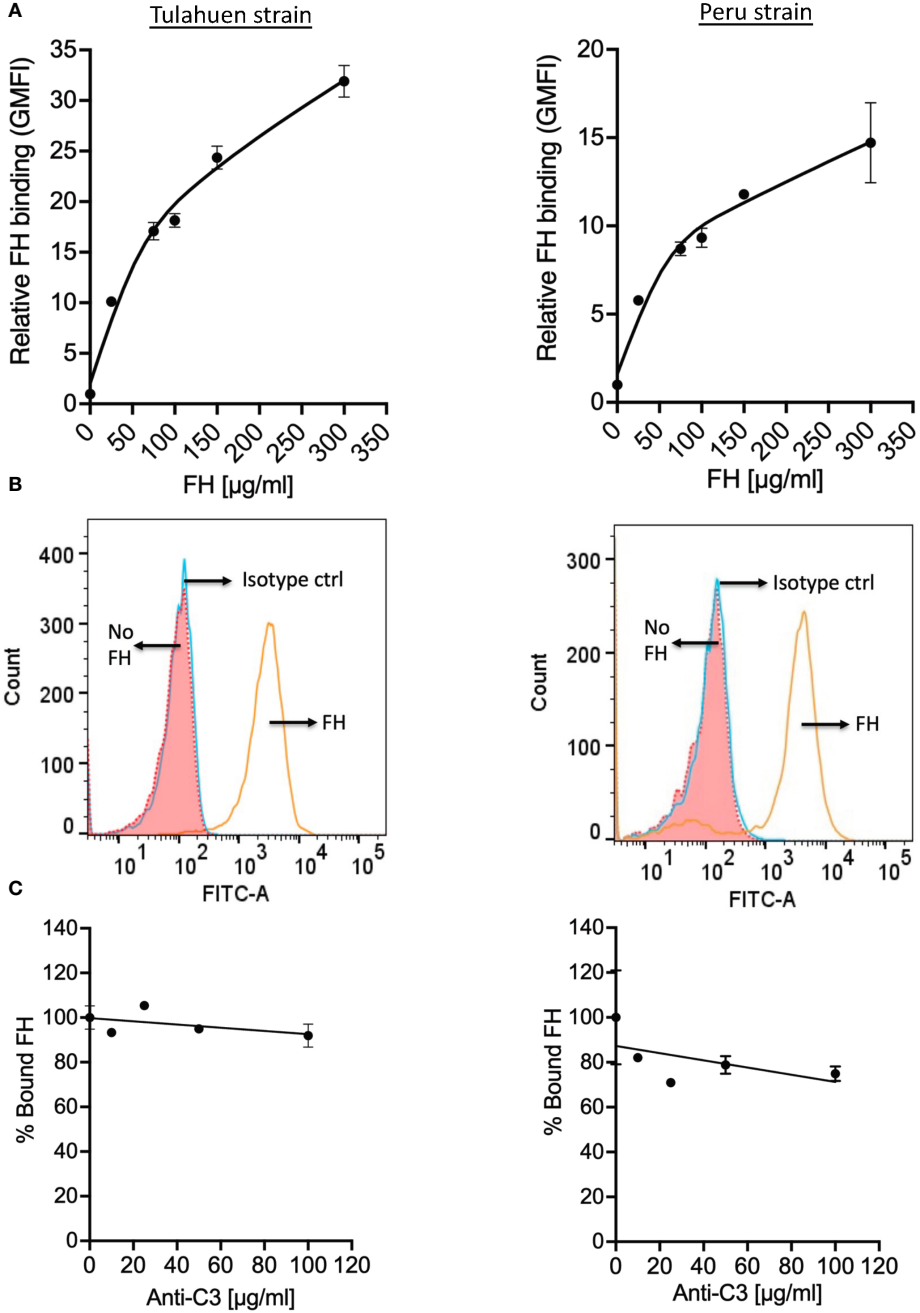
Factor H binds to trypomastigotes directly, in the absence of complement activation. 3x10^6^ Tulahuen and Peru strain trypomastigotes were incubated in 0.2% BSA/HBSS^++^ with **(A)** varying concentrations of Factor H (FH) for 30 minutes at 37°C and with **(C)** varying concentrations of polyclonal anti-C3 Ab for 30 minutes at 37°C followed by incubation with 25 μg/ml FH, at 37°C for 30 minutes. FH binding was determined by FACs as described in section 2.5 and was plotted as relative to background for **(A)** and plotted as % bound FH relative to 0 μg/ml anti-C3 (100%) for **(C)**. The data shown for **(A)** was representative of 2 independent experiments and was graphed as the mean and standard deviation of duplicates. **(B)** Histogram of FH binding at 300 μg/ml FH, as compared to the negative controls from **(A)**. The data shown for **(C)** for both strains were representative of 2 independent experiments and were graphed as mean and standard deviation of duplicates.

Trypomastigotes also bind FH in the presence of complement protein opsonization (34, 35). To assess whether trypomastigotes bound FH independently of C3 deposition, trypomastigotes were incubated with increasing concentrations of pure FH, and we observed FH binding to trypomastigotes of both strains in a dose-dependent manner (**Figures 2A, B**). FH binding to the trypomastigotes in the presence of 25 µg/ml pure FH protein with varying concentrations of polyclonal anti-C3 Ab was also tested in order to rule out the involvement of C3 fragments that may be present in the parasite culture media (e.g., in FBS or from fibroblasts). No dose-dependent significant inhibition of FH binding was observed (**Figure 2C**), indicating FH binds to trypomastigotes directly.

### 3.2 Factor H fragments rH 5-7 and rH 6-8 individually compete with Factor H for binding to trypomastigotes, but do not increase susceptibility of trypomastigotes to AP-mediated killing

**Figure 3:**
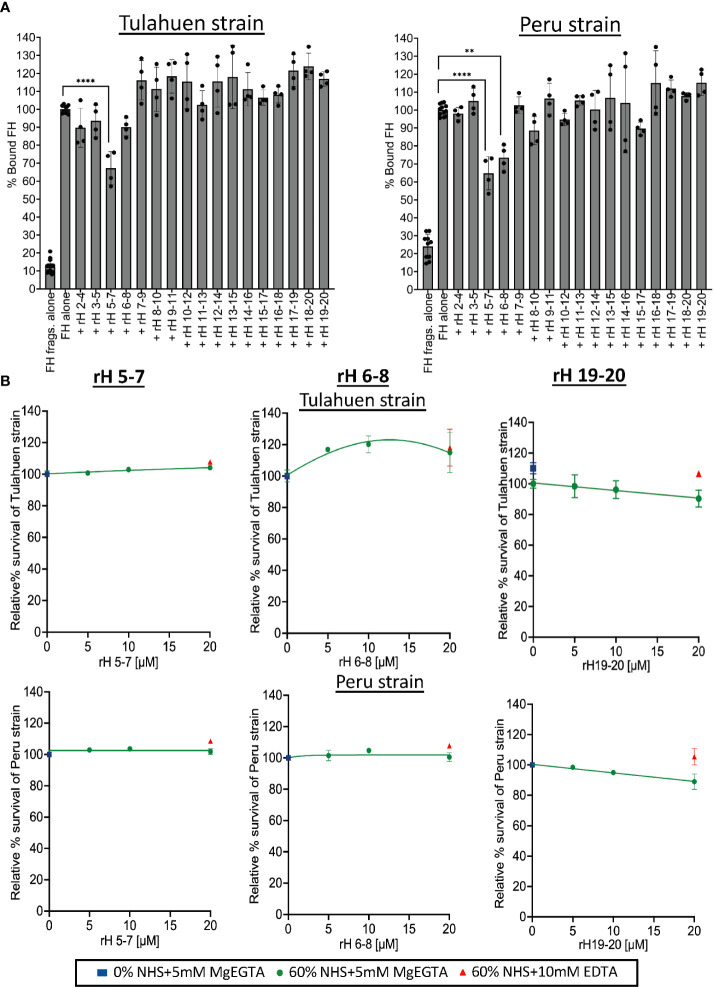
Assessment of Factor H individual 3 domain fragments in their ability to inhibit FH binding and increase susceptibility of Tulahuen and Peru strain trypomastigotes to alternative pathway-mediated killing. **(A)** 3x10^6^ Tulahuen and Peru strain trypomastigotes were incubated in 0.2% BSA/HBSS^++^ with 5 μM of overlapping 3 domain recombinant fragments of Factor H (rH), spanning regions 2-20 for 30 minutes at 37°C followed by incubation with 32 nM of Factor H (FH) at 37°C for another 30 minutes. Negative control with FH fragments and parasites alone (no FH) was added. FACS was carried out and FH binding was determined as described in section 2.5 and graphed. FH binding was determined by FACs and plotted as % bound FH relative to FH alone (100%). **(B)** 5x10^5^ Tulahuen strain and Peru strain trypomastigotes were incubated at 37°C for 60 minutes with varying concentrations of rH 5-7, rH 6-8, or rH 19-20 and 0 or 60% NHS under AP conditions (NHS + 5mM Mg EGTA) or under inactive complement conditions (NHS + 10mM EDTA). 400 μl of cold media was added and % survival of the parasites was determined as described in 2.6. The results for **(A)** were graphed as mean and standard deviation of duplicates of 2 independent experiments. Significant differences in % bound FH for samples with FH fragments were assessed by one-way ANOVA with Tukey’s multiple comparison test. Only significant reduction in the presence of FH fragments as compared to FH alone samples are shown; p<0.0001 ****, p<0.01 **, p≥ 0.05 non-significant (not shown). All comparisons to the negative control were significant; p<0.0001 **** (not shown). For **(B)**, the highest and lowest doses tested on the Peru strain represent the mean and standard deviation from duplicates in two independent experiments. For the remaining doses, the data are from a single experiment. The results for the Tulahuen strain are plotted similarly, except for rH 19-20, where all doses reflect the mean and standard deviation from duplicates in two independent experiments. Survival was plotted relative to 0 μM rH fragments (100%).

We next sought to define the molecular basis of the interactions between FH and *T. cruzi.* To define the FH domains involved in the binding to trypomastigotes, we used various overlapping contiguous three-domain FH fragments (that span FH domains 2-20) to compete with full-length FH binding to the trypomastigotes (**Figure 3A**). In order to detect full-length FH, but not the FH fragments that we used as competitors, we used a murine monoclonal anti-human FH antibody specific for domain 1 of FH (47), which is not contained in any of the fragments. A negative control with just the fragments (no FH) confirmed a lack of cross reactivity. The data showed that fragment 5-7 inhibited full-length FH binding to both strains by 33-35%, while fragment 6-8 inhibited binding of FH only to the Peru strain by 26%. Despite previous data from others indicating that removal of sialic acid leads to significantly increased susceptibility of trypomastigotes to complement-mediated killing (46), FH fragments containing the sialic acid-binding domain 20 did not inhibit full-length FH binding to the trypomastigotes. Overall, these binding experiments indicate that the region of FH spanned by domains 5-8 may interact with trypomastigotes.

Previous studies have not specifically defined the relevance of FH binding in protecting *T. cruzi* from complement-mediated attack. We hypothesize that this is due to the difficulty in selectively inhibiting FH-function on the parasite surface (e.g., using an inhibitory monoclonal antibody against the N-terminus) without also inhibiting the function of FH in the fluid phase, which would lead to immediate complement consumption. We previously overcame this obstacle by competitively inhibiting the binding of FH to cell surfaces with FH fragments that lack the N-terminal functional domains (39, 40, 48). The role of FH in protecting trypomastigotes was assessed by treating trypomastigotes with NHS under AP-only conditions in the presence of varying concentrations of the three-domain FH fragments in an attempt to compete out full-length FH binding (**Figure 3A**). Despite partial inhibition of FH binding by the 5-7 fragment for both strains (**Figure 3A**), rH 5-7 alone did not increase susceptibility of trypomastigotes to AP-mediated killing, (100% survival; **Figure 3B**). Similarly, rH 6-8 that partially inhibited FH binding inhibition to Peru trypomastigotes (**Figure 3A**) also did not increase susceptibility of trypomastigotes to the AP (**Figure 3B**). Even though no competition with FH binding was observed with rH 19-20 in the binding experiments (**Figure 3A**), given that trypomastigotes hijack host sialic acids that play a role in their resistance to complement (38, 46, 49), fragments containing the FH sialic acid-binding domain 20, rH 19-20 (**Figure 3B**) and rH 18-20 (**Supplementary Figure 3**), were also tested. However, neither fragment increased susceptibility of trypomastigotes to the AP (>90% survival; **Figure 3B**).

**Figure 4:**
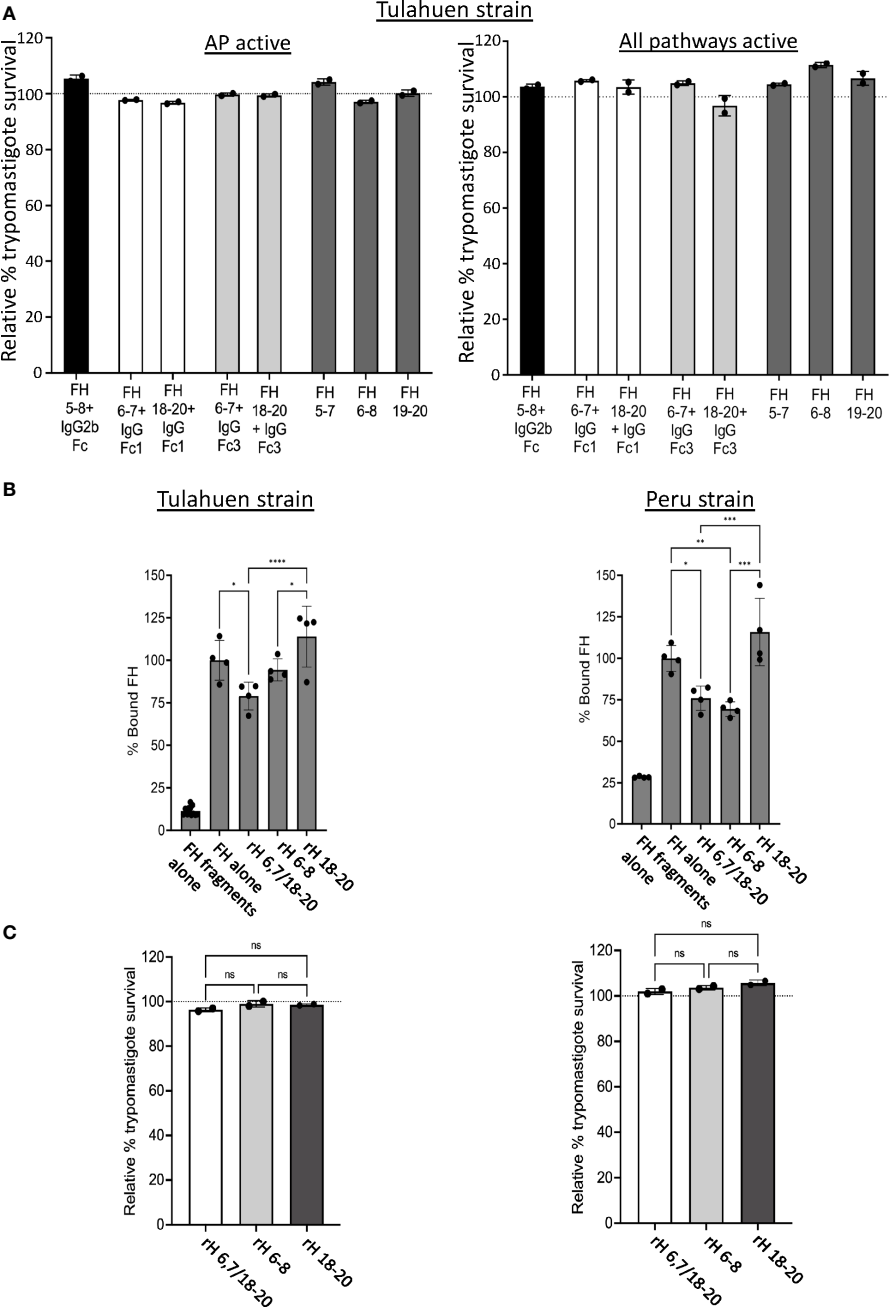
Assessment of various Factor H recombinant proteins in trypomastigote binding and alternative pathway-mediated killing. 5x10^5^ Tulahuen strain trypomastigotes were incubated at 37°C for 45 minutes with: **(A)** 20 μM of Factor H (FH)-Ig fusion proteins (i.e., human 5-8 + mouse IgG2b (black bar), human FH 6,7/18-20 + human IgGFc1 (white bars), human FH 6,7/18-20 + human IgGFc3 (light grey bars) or recombinant FH domains (rH) 5-7, rH 6-8 or rH 19-20 (dark grey bars) followed by incubation with 60% NHS under AP conditions (NHS + 5mM Mg EGTA) or when all 3 complement pathways active (NHS only) at 37°C for 60 minutes; **(C)** 5 μM of FH 6,7/18-20, rH 6-8 and rH 18-20 followed by incubation with 60% NHS under AP conditions (NHS + 5mM Mg EGTA) at 37°C for 60 minutes. 400 μl of cold media was added and % survival of the parasites was determined as described in section 2.6, and survival was plotted relative to 0 μM rH fragments or fusion proteins (100%, dotted line). **(B)** 3x10^6^ Tulahuen or Peru strain trypomastigotes were incubated in 0.2% BSA/HBSS^++^ with 5 μM of recombinant Factor H (rH) 6,7/18-20, rH 6-8 or rH 18-20 for 30 minutes at 37°C followed by incubation with 32 nM of FH at 37°C for 30 minutes. Negative control with FH fragments and parasites alone (no FH) was added. FH binding was assessed by FACs as described in section 2.5 and plotted as % bound FH relative to FH alone (100%). For **(A)** and **(C)**, the results are representative of 2 independent experiments and were graphed as mean and standard deviation of duplicates. For **(B)**, results were graphed as mean and standard deviation of duplicates of 2 independent experiments. Significant differences in % bound FH for samples with FH fragments were assessed by one-way ANOVA with Tukey’s multiple comparison test; p<0.0001 ****, p<0.001 ***, p<0.01 **, p≥ 0.05 non-significant (not shown). All comparisons to the negative control were significant; p<0.0001 **** (not shown). *, p<0.05.

In order to confirm the results obtained with the individual fragments, recombinant fusion proteins encompassing FH domains 5-8 fused to mouse IgG2b or FH region 6-7 or 18-20 fused to human IgG Fc1 or human IgG Fc3 were tested in the survival assay. rH 5-7, rH 6-8 and rH 19-20 fragments were used as controls. None of the fusion proteins tested were able to effectively compete with FH to increase AP-mediated or intact complement-mediated killing (**Figure 4A**) of trypomastigotes. Altogether, the survival assay results indicate none of the individual FH fragments containing two to four contiguous FH domains alone is sufficient for FH to effectively bind to and protect trypomastigotes.

### 3.3 Factor H 6-7-18-20 protein partially decreases FH binding to trypomastigotes, but does not increase AP-mediated killing of trypomastigotes

Based on the FH binding and survival assay results with the individual 3 domain fragments, we hypothesized that multiple regions of FH may simultaneously interact with and protect trypomastigotes from complement. To test this hypothesis, a FH recombinant fragment that contains two of the three previously described polyanion-binding FH regions, domains 6 and 7 and domains 18-20 (rH 6,7/18-20), was assessed for its ability to compete with full-length FH binding to the parasite and increase trypomastigote killing by the AP. rH 6,7/18-20 decreased FH binding by 21-24% for both trypomastigote strains, a level similar to that obtained with rH 6-8 that was included as a control (**Figure 4B**). rH 6,7/18-20 was also assessed in its ability to increase AP-mediated killing of trypomastigotes, but the survival was >90% for both the strains, which was again similar to FH fragments rH 6-8 and rH 18-20, tested as controls (**Figure 4C**).

Two observations make the C-terminus of FH a top candidate for being central to FH binding to trypomastigotes: (a) the C-terminus of FH contains the only sialic acid-binding domain of FH (i.e., domain 20) (32, 33) and is essential for binding to several cell surfaces by binding to both sialic acid or other polyanions in combination with C3 fragment deposits (50) and (b) trypomastigotes sequester host sialic acid to their surface and removal of the sialic acid causes parasites to succumb to complement attack. However, our data thus far did not support this notion. Although all recombinant proteins used in this study have been used in other studies (39, 40, 51), we sought to confirm that the recombinant proteins that encompass the C-terminus were functional (i.e., rH 18-20, rH 19-20, and rH 6,7/18-20). Epimastigotes, which bind FH in a C3-dependent manner (**Figure 1**) were exposed to 10% NHS that causes 50% AP-mediated killing (**Supplementary Figure 1**), in the presence or absence of varying doses of rH 18-20, rH 19-20, and rH 6,7/18-20 (**Supplementary Figure 4**). The data show that all proteins tested reduced the survival of epimastigotes by 65-75%, in a dose-dependent manner, and indicate that (a) epimastigotes, although susceptible to complement, are still partially protected by FH, (b) that the C-terminus of FH is important for FH protection of epimastigotes, and (c) the recombinant proteins rH 18-20, rH 19-20 and rH 6,7/18-20 all contain binding sites for epimastigotes and are functional.

### 3.4 Factor H-related protein 5 can compete and inhibit binding of Factor H to the trypomastigotes, conferring resistance to all 3 complement pathways

**Figure 5:**
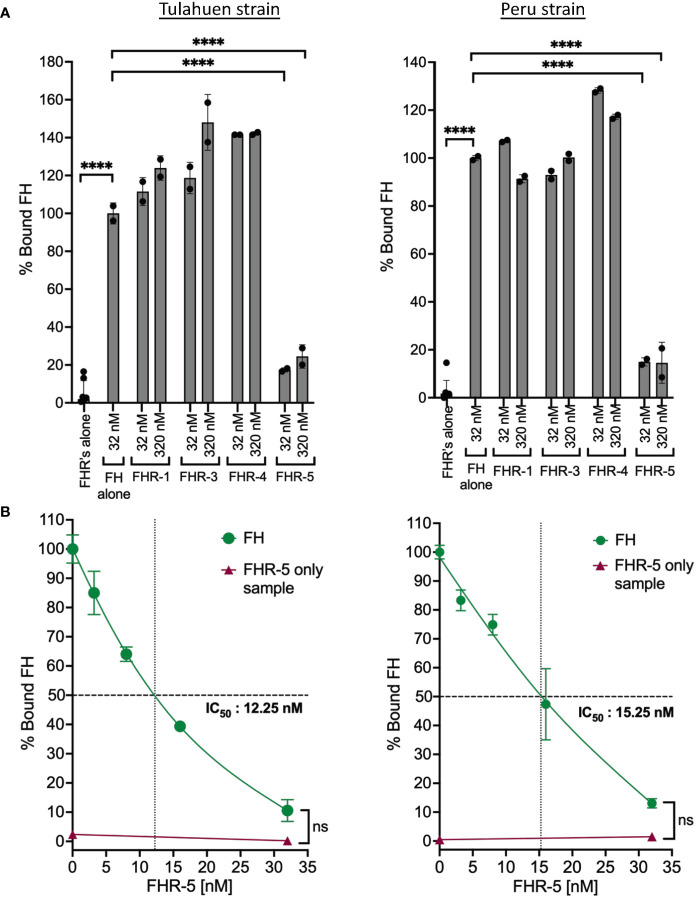
Factor H-Related Protein-5 completely inhibits the binding of Factor H to trypomastigotes. 3x10^6^ Tulahuen or Peru strain trypomastigotes were incubated in 0.2% BSA/HBSS^++^ with: **(A)** Factor H-related proteins (FHRs): FHR-1, FHR-3, FHR-4 and FHR-5 and **(B)** varying concentrations of FHR-5 for 30 minutes at 37°C followed by incubation with 32 nM of FH at 37°C for 30 minutes. Factor H (FH) binding was determined by FACs and plotted as % bound FH relative to FH alone (100%). The data for **(A)** and **(B)** were representative of 2 independent experiments and were graphed as mean and standard deviation of duplicates. For **(A)** significant differences in % bound FH for samples with FHRs as compared to FH alone samples were assessed by one-way ANOVA with Tukey’s multiple comparison test. Only significant reduction in the presence of FHRs as compared to FH alone samples are shown; p<0.0001 ****, p≥ 0.05 non-significant (not shown). For **(B)**: only p≥ 0.05 non-significances were shown.

FHRs are found in serum at low concentrations (i.e., FHR-1: ~2-15 μg/ml, FHR-2: ~3 μg/ml, FHR-3: 0.81 μg/ml, FHR-4: 2.55± 1.46 μg/ml, FHR-5: ~3–6 μg/ml) that are several-fold lower than FH (52, 53). These proteins contain domains with high sequence identity to the polyanion- and C3b-binding regions of FH (53). However, unlike FH, FHRs lack the complement regulatory domains 1-4 and thus cannot protect surfaces from complement attack. The shared sequence identity allows FHRs to bind to FH ligands (described for FHR-1, FHR-3, FHR-4 and FHR-5) such as C3 fragments and polyanions on surfaces and are proposed to act as deregulators by competing with FH for binding to surfaces, thus inhibiting FH negative regulatory activity (53). It has been posited that FHRs may have evolved to divert FH away from pathogen surfaces (54). We therefore hypothesized that FHR proteins may outcompete FH binding to the parasite. For this, the abilities of FHRs 1, 3, 4 and 5 (**Supplementary Figure 5**) to compete with FH to bind to the trypomastigotes was tested at conditions where the molar concentration of the FHRs was the same as FH (32 nM) or 10x more than the concentration of FH (320 nM). The data showed that FHRs 1, 3 and 4 that contain regions with sequence identity to two FH polyanion-binding domains (**Supplementary Figure 5**) were not able to compete with FH binding to trypomastigotes of either strain. However, FHR-5 that contains regions that shared sequence identity with all three FH polyanion-binding domains (**Supplementary Figure 5**) significantly inhibited the binding of FH to both strains by ~80-90% (**Figure 5A**). The data in **Figure 5B** indicates inhibition of FH binding by FHR-5 was dose-dependent and achieved complete inhibition at the highest dose tested (32 nM), with an IC_50_ of 12.25 nM for Tulahuen and 15.25 nM for Peru. Overall, these results suggest *T. cruzi* interacts with FH through all three FH polyanion-binding domains.

**Figure 6:**
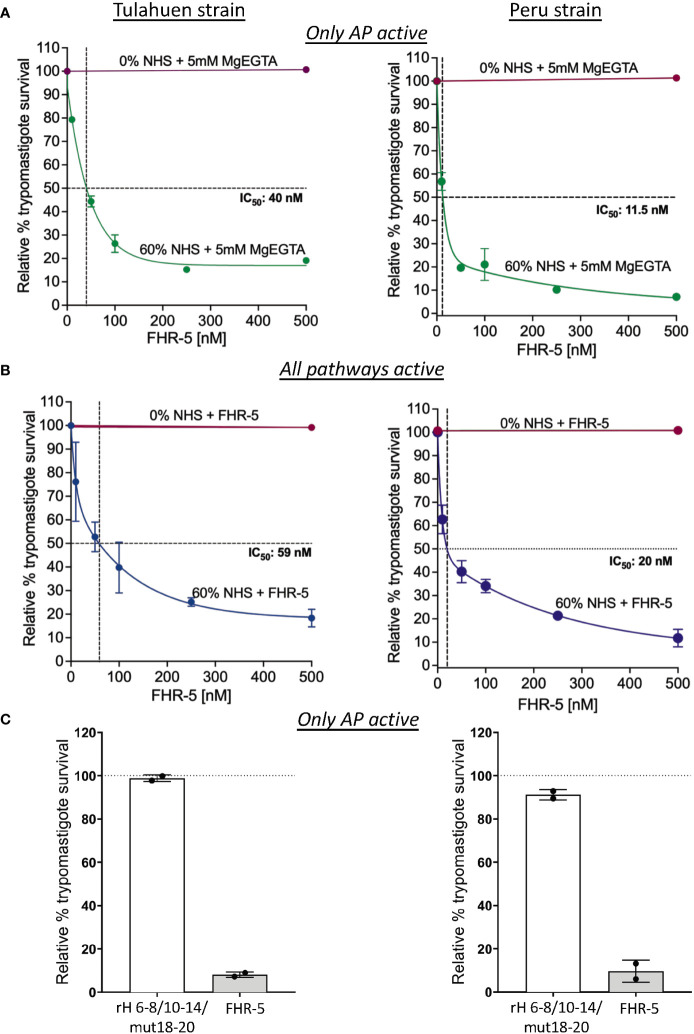
Assessment of FHR-5 and Factor H 6-8/10-14/18-20 with a D1119G mutation on trypomastigote susceptibility to complement-mediated killing. 5x10^5^ Tulahuen strain or Peru strain trypomastigotes were incubated at 37°C for 45 minutes with **(A)** and **(B)** varying concentrations of Factor H-related protein-5 (FHR-5) and **(C)** 500 nM of Factor H 6-8/10-14/18-20 with a D1119G mutation (rH 6-8/10-14/mut18-20) or FHR-5. For **(A)** and **(C)**, samples are incubated then with 60% NHS under AP conditions (NHS + 5mM Mg EGTA) and for **(B)** when all 3 complement pathways are active. Samples treated with 0% NHS were added as controls. 400 μl of cold media was added and % survival of the parasites was determined as described in section 2.6. Survival was plotted relative to 0 μM FHR-5 (100%) for **(A)** and **(B)** and relative to 0 μM rH 6-8/10-14/mut18-20 (100%) for **(C)**. For **(A)**, the data for Tulahuen strain were representative of 3 independent experiments and the data for Peru strain was representative of 2 independent experiments, and both were graphed as mean and standard deviation of duplicates. For **(B)**, the data for both strains were graphed as mean and standard deviation of duplicates of 2 independent experiments. For **(C)**, the data for both strains were graphed as mean and standard deviation of duplicates of an independent experiment.

We took advantage of the ability of FHR-5 to inhibit the binding of FH to the trypomastigotes to assess the consequences of blocking FH binding on parasite survival against complement-mediated attack. Varying concentrations of FHR-5 were incubated with NHS and trypomastigotes under AP conditions or when all three pathways were active, and survival was assessed (**Figures 6A, B**). The data showed that survival was reduced to ~19% for the Tulahuen strain with an IC_50_ of 40 nM and to ~7% for the Peru strain with an IC_50_ of 11.5 nM (**Figure 6A**) under AP-only conditions. The survival was also reduced to ~18% for the Tulahuen strain with an IC_50_ of 59 nM and to ~12% for the Peru strain with an IC_50_ of 20 nM (**Figure 6B**) when all pathways were active. Overall, these data indicate that inhibiting FH binding to previously resistant trypomastigotes renders them significantly susceptible to complement-mediated killing.

### 3.5 Recombinant Factor H 6-8/10-14/18-20 with a D1119G mutation (rH 6-8/10-14/mut18-20) does not increase AP-mediated killing of trypomastigotes

Increased killing of the trypomastigotes by FHR-5 suggests that all 3 FH polyanion-binding regions are important for FH binding to the trypomastigotes. However, even though FHR-5 contain domains with high sequence identity to FH, the domains of FHR-5 are not strictly identical to FH domains. To determine if a FH protein containing all 3 FH polyanion-binding regions can compete with full-length FH and increase killing of the trypomastigotes, we generated a FH recombinant protein, rH 6-8/10-14/mut18-20 with a point mutation in domain 19 (D1119G). This mutation completely impairs the ability of the C-terminal FH region to bind to C3b, but not to polyanions found on human cell surfaces (51). A FH protein with the D1119G mutation was also shown to bind to the highly sialylated *Neisseria* gonococcal surface and increase killing in a similar manner as a FH protein containing wildtype 19-20 region (55). We therefore hypothesized that rH 6-8/10-14/mut18-20 would compete with full-length FH for binding to the highly sialylated trypomastigote surface and increase killing of the trypomastigotes; however, when tested, the survival was >89% for both the strains (**Figure 6C**) indicating that rH 6-8/10-14/mut18-20 was not able to compete with FH. FHR-5 was added as control and showed increased killing of the trypomastigotes >90% as expected. The data suggests that the D1119G mutation in rH 6-8/10-14/mut18-20-Fc or the sequence differences with FHR-5 may lead to lack of competition of rH 6-8/10-14/mut18-20-Fc with FH.

## 4 Discussion

Evasion of the complement system is an important prerequisite for pathogens to establish infection in the host. *T. cruzi* have developed various complex strategies to escape complement-mediated attack that include expression of complement binding molecules on the trypomastigote surface (e.g., *T. cruzi* calreticulin, trypomastigote decay-accelerating factor) and inducing micro vesicles that inhibit complement activation by interacting with C3 convertases (7). Although many pathogens have been identified as using FH, the negative regulator of complement, as an evasion strategy to protect from the AP of complement and the specific mechanisms involved have been defined, work remains to be done in the case of *T. cruzi*. Hijacking FH is also thought to be a potential strategy adopted by *T. cruzi.* Studies have shown that FH binds to culture-derived metacyclic trypomastigotes and epimastigotes that are pre-opsonized with C3b, with higher affinity to the trypomastigotes (34, 35). Here, we delved into the mechanisms involved in the interaction between FH and *T. cruzi* and the effect on parasite survival once FH binding is thwarted. The data indicate that even though FH bound to both Brazil strain epimastigotes (complement-susceptible, **Supplementary Figure 1**) and Tulahuen and Peru strain trypomastigotes (complement-resistant, **Supplementary Figures 2A, B**), direct FH binding in the absence of complement opsonization was observed only for the trypomastigote strains (**Figures 1A** and **2**). Thus, FH bound to epimastigotes in a C3-dependent manner (**Figures 1B, C** and **Supplementary Figure 4**), while FH bound directly to live trypomastigotes in a C3-independent manner (**Figure 2C**).

*T. cruzi* cannot synthesize sialic acid *de novo* and relies on a trans-sialidase enzyme to sequester host α2, 3-linked sialic acids for placement on mucins present on the parasite surface (36, 37). Given that FH contains a sialic acid-binding domain in the C-terminus (i.e. domain 20) (32, 33), the process of hijacking FH by *T. cruzi* is hypothesized to occur via this FH domain, similar to other pathogens, such as *N. gonorrhoeae* (21). This hypothesis has not been directly addressed, but is supported by reports showing resistant trypomastigotes being partially susceptible to complement-mediated lysis after treatment with sialidase (38, 46). Interestingly, both studies indicated that sialic acid alone does not explain the resistance of these parasites to complement-mediated lysis. Our domain mapping studies, and survival assays also indicate that sialic acid may not be the sole contributor of FH binding to the trypomastigotes. Fragments rH 18-20 and rH 19-20 containing the sialic acid binding domain 20, which effectively compete with full-length FH for binding to human cells (39, 51), did not compete with full-length FH for binding to trypomastigotes (**Figure 3**). Additionally, survival assays using rH 18-20 (**Supplementary Figure 3**) and rH 19-20 (**Figure 3B**) did not cause increased susceptibility of the trypomastigotes to AP-mediated lysis. Thus, it is possible that this sialic acid binding domain is not sufficient as previously hypothesized, but that additional FH domains may be required for binding to other unknown ligands on the parasite.

Our domain mapping studies using individual fragments showed that a fragment containing domain 7 (a well-established polyanion-binding FH region), rH 5-7, partially competed with full-length FH and decreased FH binding to both strains of the trypomastigotes (**Figure 3A**). rH 6-8, which also contains domain 7, also decreased FH binding, but only to the Peru strain trypomastigotes. However, despite having partially inhibited FH binding, fragments rH 5-7 and rH 6-8, similar to the domain 20 fragments, did not cause increased susceptibility of the trypomastigotes to AP-mediated lysis (**Figure 3B**). Given these results, we sought to test a different source of these domain fragments that consist of fusion proteins containing 3 to 4 domains each of human FH (5-8, 6-8 or 18-20) fused to the Fc-region of human or mouse IgG. The advantage of these fusion proteins is that if the FH fragment portion of the protein competes with FH and binds to the parasite, the AP will be activated due to decreased FH regulation, while the Fc portion of the fusion protein will activate the CP. This may increase the susceptibility of the parasites to complement-mediated lysis to a detectable level. As examples, a fusion protein containing FH domains 6-7 and IgG Fc was effective in rodent models of *Neisseria meningitidis, N. gonorrhoeae*, group A streptococci and non-typeable *Haemophilus influenzae* (56–59). However, in the *T. cruzi* model, the fusion proteins did not increase susceptibility to complement-mediated lysis (**Figure 4A**). Overall, the results with individual FH fragments and fusion proteins support the notion that more than one region of FH is involved in binding to the parasite and therefore individual fragments tested cannot out-compete full-length FH. To begin to address this, rH 6,7/18-20, a recombinant protein that contains two of the three previously described polyanion FH binding regions, was tested as a competitor and only inhibited FH binding to trypomastigotes by 21-24%, a level similar to that observed with rH 6-8, without any additive inhibitory effect due to the 18-20 region (**Figure 4B**). Additionally, rH 6,7/18-20 had no effect on the survival of the trypomastigotes (**Figure 4C**).

FHR proteins belongs to the FH family of proteins that circulate in blood at low concentrations and have domains with significant sequence identity to FH, but lack domains homologous to FH domains 1–4, which are responsible for the complement regulatory function (60). Most FHRs share sequence identity only to FH domains 6-8 and 19-20. An exception is FHR-5 that also contains domains with significant sequence identity to domains 10-14 of FH and thus shares identity with all 3 known polyanion-binding domains of FH. The shared sequence identity allows FHRs (FHR-1, FHR-3, FHR-4 and FHR-5) to bind to FH ligands such as C3 fragments and polyanions on surfaces, resulting in a FH antagonistic function termed “complement deregulation”. Similar to FH, FHRs can also be hijacked and bound by pathogens, even though the advantage of binding FHRs is not clearly understood (54). FHRs are considered to be decoys that compete with FH in binding to common ligands on pathogen surfaces and are proposed to increase opsonization of microbes, dying cells, and cellular debris, and increase inflammation. We took advantage of the sequence identity of the polyanion-binding regions and the shared ability to bind to common ligands between FH and FHRs to use them as a tool to investigate in detail the interaction of FH with *T. cruzi.* FHR-1, FHR-3 and FHR-4 that contain a combination of two of the three known polyanion-binding FH regions did not compete with full-length FH in binding to the parasites (**Figure 5A**). FHR-1 has >95% identity with domains 18-20 of full-length FH (**Supplementary Figure 5**). The inability of FHR-1 to compete with FH and inhibit FH binding to trypomastigotes serves as yet another line of evidence that domains 19-20, which contain the only known sialic acid-binding domain of FH, is not sufficient for binding of FH to trypomastigotes either on its own, or in concert with domains homologous to FH 6-7. Interestingly, FHR-5, that has regions with sequence identity to all three described polyanion-binding regions of FH, competed with FH and resulted in complete inhibition of binding to the parasite when the concentration of FH and FHR-5 was similar, supporting the notion that FH most likely binds to *T. cruzi* through all 3 polyanion-binding domains (**Figures 5A, B**). We speculate that the ability to bind FH through three distinct regions may be an evolutionary mechanism whereby trypomastigotes may prevent FHRs (with the exception of FHR-5) from diverting FH away from the parasite surface. Our data have also identified a novel way to completely inhibit FH binding to the *T. cruzi* surface, thereby elucidating a role for FH in protecting trypomastigotes from complement-mediated killing, evidenced by a marked reduction in parasite survival when FHR-5 was added to NHS (**Figures 6A, B**). This is the first evidence showing how critical the protection by FH is for survival of *T. cruzi* and suggests that it may be the most or one of the most important individual complement regulators on the parasite surface. Residual survival of trypomastigotes in the presence of FHR-5 was similar when only the AP was active or when all three pathways were intact, suggesting that the parasite can still partially protect itself from lysis due to other complement evasion mechanisms specific for the CP (51, 61–63). It is worth noting that at the concentrations present in NHS, endogenous FHR-5 cannot outcompete FH on trypomastigotes because they are fully resistant to even 60% NHS. Killing is only seen when the trypomastigotes were treated with FHR-5 and NHS (**Figures 6A, B**). During an infection, the ability of FHR-5 to compete with FH for binding to the parasites may occur if FHR-5 levels in the serum or cellular microenvironments increases. Future studies aimed at understanding the specific role of FHR-5 in the context *T. cruzi* infection are warranted. Recent work by Lavender and colleagues showed that FHR-5 bound to the major outer membrane protein (PorB) of the pathogenic Neisseriae, but only when lipooligosaccharide was sialylated, invoking a cooperative binding mechanism as was shown between the C-terminus of FH and host cell-associated sialic acid and C3b (64). Similar to our findings, addition of FHR-5 to NHS enhanced Neisserial killing (64), which lends support to the theory that FHRs may have evolved as decoy to counteract pathogens co-opting FH.

Even though results with FHR-5 supported the importance of all 3 polyanion-binding regions in binding to the trypomastigotes, the domains of FHR-5 have certain sequence differences with FH. We generated rH 6-8/10-14/mut18-20-Fc protein, that contains human FH sequences for domains 6-8 and 10-14 and a mutant C terminal region (D1119G). The D1119G mutation, unable to bind to C3b, significantly reduces the ability of domains 19-20 of FH to bind to human cell surfaces (51), but does not impair FH binding to high sialic acid content surfaces, such as found in *N. gonorrhoeae* (55). On gonococcal surface, a D1119G-modified FH protein was found to bind in a similar manner to a FH protein containing wildtype 19-20 region. Given the high sialylation of trypomastigotes and the similarity of the binding of D1119G 19-20 mutant and wildtype 19-20 to sialylated surfaces, we expected rH 6-8/10-14/mut18-20-Fc protein to compete with full-length FH and increase AP-mediated killing of trypomastigotes. Unexpectedly, rH 6-8/10-14/mut18-20-Fc D1119G mutation did not increase parasite susceptibility to complement (**Figure 6C**). These results suggest two possibilities (i) in *T. cruzi*, the D1119G mutation impairs the function of the 19-20 region of the FH protein in binding to the trypomastigotes, mimicking a protein with only the 6-8/10-14 regions, supporting our other data that indicates all three FH polyanion-binding regions are critical for FH binding to trypomastigotes; (ii) the D1119G mutation does not impair the function of the 19-20 region for binding to trypomastigotes, but the differences in the sequences within FH polyanion-binding regions (6-8, 10-14, and 19-20), between FH and FHR-5, may explain the lack of competition of rH 6-8/10-14/mut18-20-Fc. Future studies aimed at determining whether these sequence differences affect FH binding to parasites are warranted.

The involvement of all three polyanion domains would also suggest the existence of a potential FH-binding protein receptor or of FH-binding polyanions other than sialic acids on the parasite surface. This notion is supported by work from Tomlinson et al. (38) that showed that the survival of the trypomastigotes was more than 90% when sialic acid is removed from the parasites and treated with NHS that is not desialylated. Conversely, Kipnis et al. (46) showed that only 32% of the parasites survive after parasite desialyation in the presence of NHS. However, as Tomlinson et al. indicated, the higher rate of decreased survival that is observed in this case might be due to the presence of proteases or azide in sialidase preparations that can increase susceptibility of trypomastigotes to complement (38) and warrants further investigation. Regardless, both works indicate that there was still a portion of parasites that are resistant to complement-mediated lysis and that the removal of surface proteins with trypsin led to 100% killing in the presence of complement (46). On host cells, FH binds to a combination of polyanions and C3b/d (51). However, in the case of trypomastigotes, FH may be binding to a combination of polyanionic markers and FH-binding receptor protein on the parasite surface, thereby removing the need for C3b/d for FH binding. A similar FH binding mechanism is proposed to occur with *N. meningitidis*, where bacterial sialic acid potentially acts as a docking station for FH, perhaps through binding CCP 20, while FH CCPs 6-7 bind to Neisserial surface protein A (NspA) (65). Future studies to identify any potential FH binding receptor(s) on *T. cruzi* trypomastigotes and the mechanisms of FH binding are underway.

It is important to note that the current data obtained are for two trypomastigote strains among many. Our study using the Tulahuen strain, which is associated with the chronic phase of the disease and the Peru strain, which is associated with the acute form of the disease, shows similar relevance of FH binding in the survival of parasite strains that are responsible for two different presentations of the disease. However, work done by Cestari et al. (66) has shown that trypomastigote strains have varying susceptibility to complement-mediated killing. Thus, studies aimed at understanding if these variations in susceptibility are related to the nature of the FH interaction with *T. cruzi* are warranted.

In conclusion, our study demonstrated that FH binds to trypomastigotes directly in a C3-independent manner and inhibition of FH-mediated protection from the AP of complement leads to killing of the parasites. This is the first study to show the critical contribution of host FH to the protection of *T. cruzi* from complement-mediated killing, to indicate that binding to sialic acid may not be the critical ligand for FH, and that multiple FH domains are involved in the interaction. These data may have implications for developing therapeutics and vaccine candidates against *T. cruzi*.

## Data availability statement

The original contributions presented in the study are included in the article/**Supplementary Material**. Further inquiries can be directed to the corresponding author.

## Author contributions

Experiments were designed by SM and VF and were conducted by SM and SE. The results were analyzed and interpreted and written into the manuscript by SM and VF. SE provided technical support and proofreading of the manuscript. GR-T provided key technical design contributions for setting up the parasite culture and survival assay experiments. KW provided the fusion proteins comprising human FH domains 18-20 or 6-7 fused to the Fc fragment of human IgG1(Fc1) or human IgG3 (Fc3). SR and JS provided rH 6,7/18-20, fusion protein comprising FH domains 5-8 fused to the Fc fragment of murine IgG2b, and rH 6-8/10-14/mut18-20 fused to the Fc fragment of human IgG1. All authors critically reviewed and contributed to the manuscript, and approved the submitted version.
