# Supplementary material for: Mechanisms by which Factor H protects Trypanosoma cruzi from the alternative pathway of complement
Source: Front Immunol. 2024 Feb 1;15:1152000. doi: 10.3389/fimmu.2024.1152000 (PMC10867245; doi:10.3389/fimmu.2024.1152000)
Supplement: Supplementary file 1 [file DataSheet_1.docx]

Supplementary Material

## Supplementary Figures


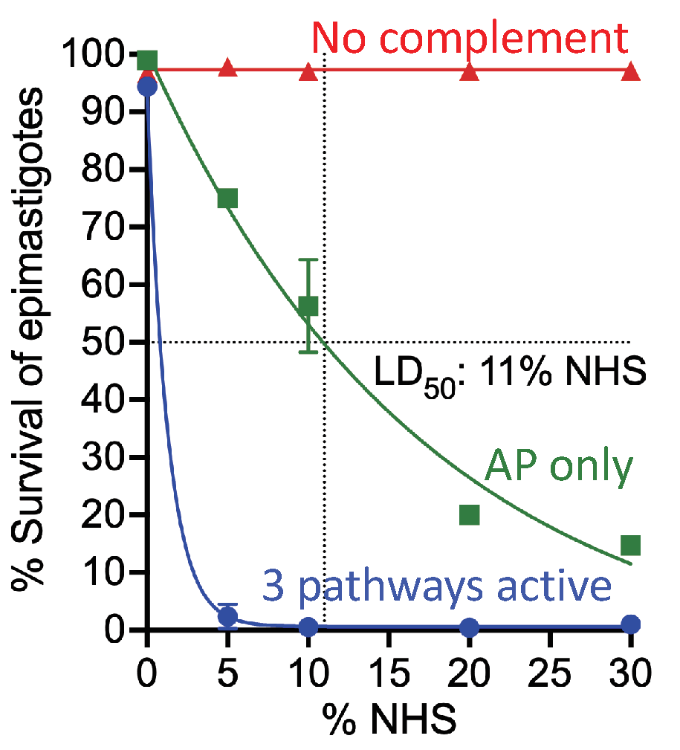


**Supplementary Figure 1.** **Brazil strain** **epimastigotes (non-infective forms) are susceptible to complement-mediated killing.** 5x10^5^ Brazil strain epimastigotes were incubated at 37°C with varying concentrations of NHS diluted with PBS^=^ under (i) AP conditions (NHS + 5mM Mg EGTA); (ii) conditions when all the 3 complement pathways were active (NHS only); (iii) and under inactive complement conditions (NHS + 10mM EDTA); for 60 minutes in a total 50 μl volume. The complement reaction was stopped by adding 400 μl of cold media and % survival of the parasites was determined as described in “Materials and Methods” (section 2.6) and graphed. The data was representative of 2 independent experiments and was graphed as mean and standard deviation of duplicate observations.


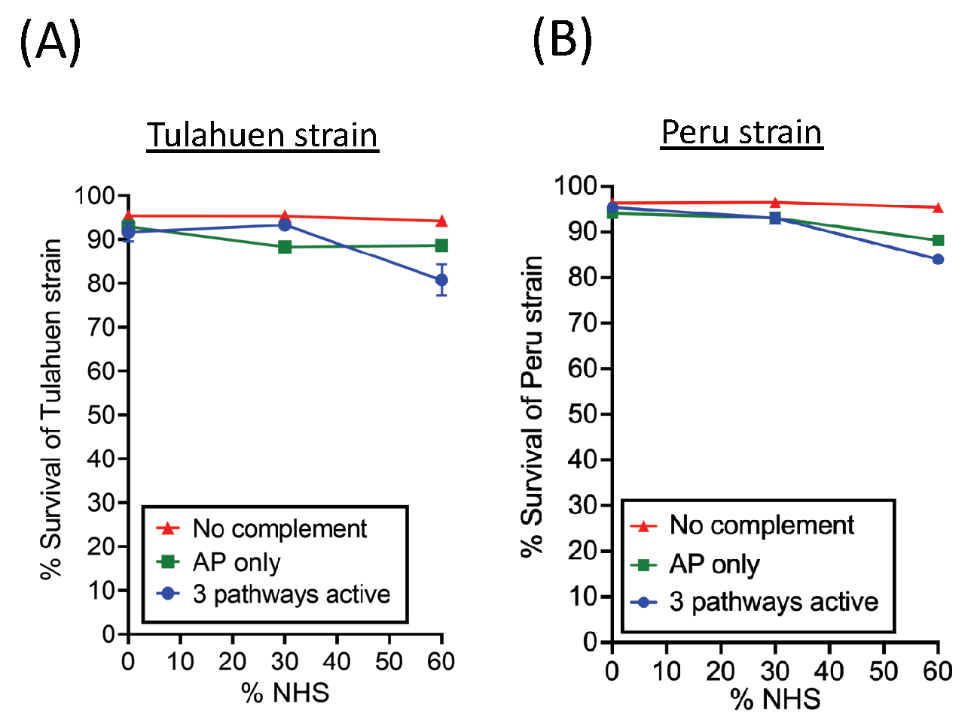


**Supplementary Figure 2.** **Tulahuen and Peru strain** **trypomastigotes (infective forms) are resistant to complement-mediated killing.** 5x10^5^ **(A**) Tulahuen strain and (**B**) Peru strain trypomastigotes were incubated at 37°C with varying concentrations of NHS diluted with PBS^=^ under (i) AP conditions (NHS + 5mM Mg EGTA); (ii) conditions when all the 3 complement pathways were active (NHS only); (iii) and under inactive complement conditions (NHS + 10mM EDTA); for 60 minutes in a total 50 μl volume. The complement reaction was stopped by adding 400 μl cold media and % survival of the parasites was determined as described in “Materials and Methods” (section 2.6) and graphed. Results represent the mean and standard deviation from two (Tulahuen) and one (Peru) independent experiments with duplicates.


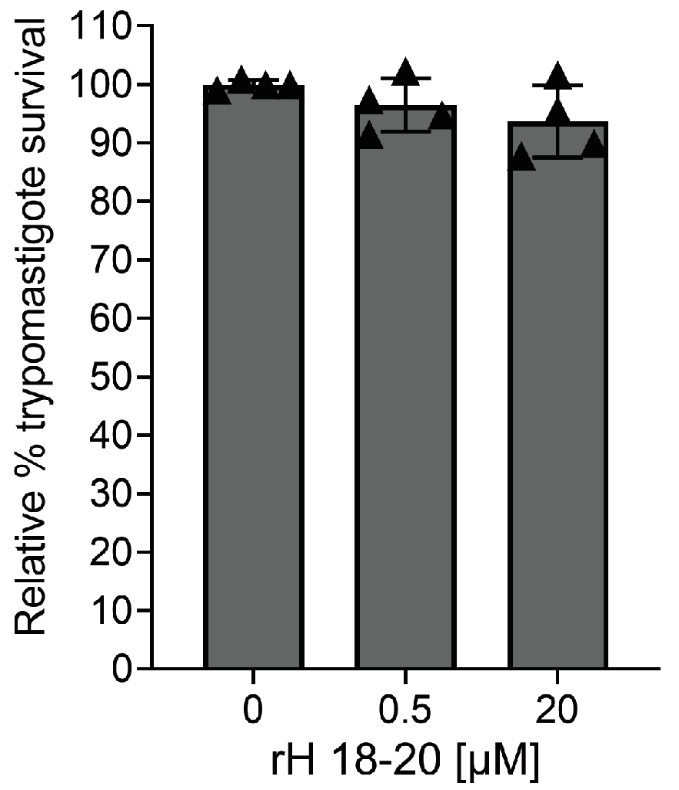


**Supplementary Figure 3**: **Factor H recombinant fragment 18-20 did not cause increased susceptibility of Tulahuen strain trypomastigotes to alternative pathway complement-mediated killing.** 5x10^5^ Tulahuen strain trypomastigotes were incubated at 37°C for 45 minutes with varying concentrations of recombinant Factor H domains 18-20 (rH 18-20) followed by incubation with 60 % NHS under AP conditions (NHS + 5mM Mg EGTA) at 37°C for another 60 minutes in a total 50 μl volume. The complement reaction was stopped by adding 400 μl of cold media, % survival of the parasites was determined as described in “Materials and Methods” (section 2.6), and survival was plotted relative to 0 μM rH 18-20 (100 %). The data shown were graphed as mean and standard deviation of duplicates from 2 independent experiments. Significant differences in relative % survival for samples were assessed by one-way ANOVA with Tukey’s multiple comparison test; p≥ 0.05 non-significant (ns).


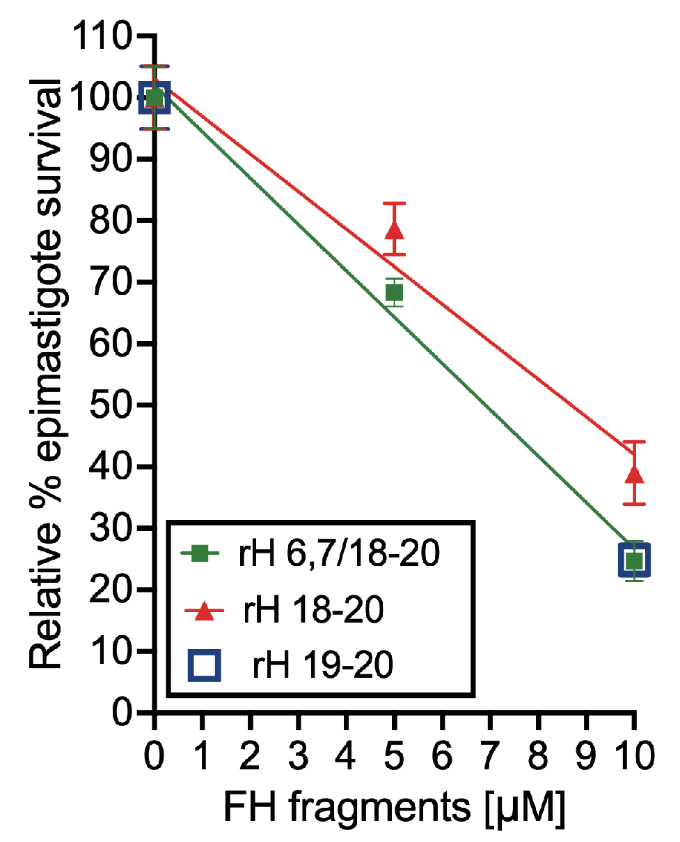


**Supplementary Figure 4**: **Factor H recombinant proteins rH 18-20, rH 19-20 and rH 6,7/18-20 increased susceptibility of Brazil strain epimastigotes to alternative pathway-mediated killing.** 5x10^5^ Brazil strain epimastigotes were incubated at 37°C for 45 minutes with varying concentrations of Factor H (FH) 6-7-18-20 and recombinant (rH) 18-20 and rH 19-20 followed by incubation with 60 % NHS under AP conditions (NHS + 5mM Mg EGTA) at 37°C for 60 minutes in a total 50 μl volume. The complement reaction was stopped by adding 400 μl of cold media and % survival of the parasites was determined as described in “Materials and Methods” (section 2.6) and survival was plotted relative to 0 μM FH fragments (100 %). The data was representative of 2 independent experiments and were graphed as mean and standard deviation of duplicates.


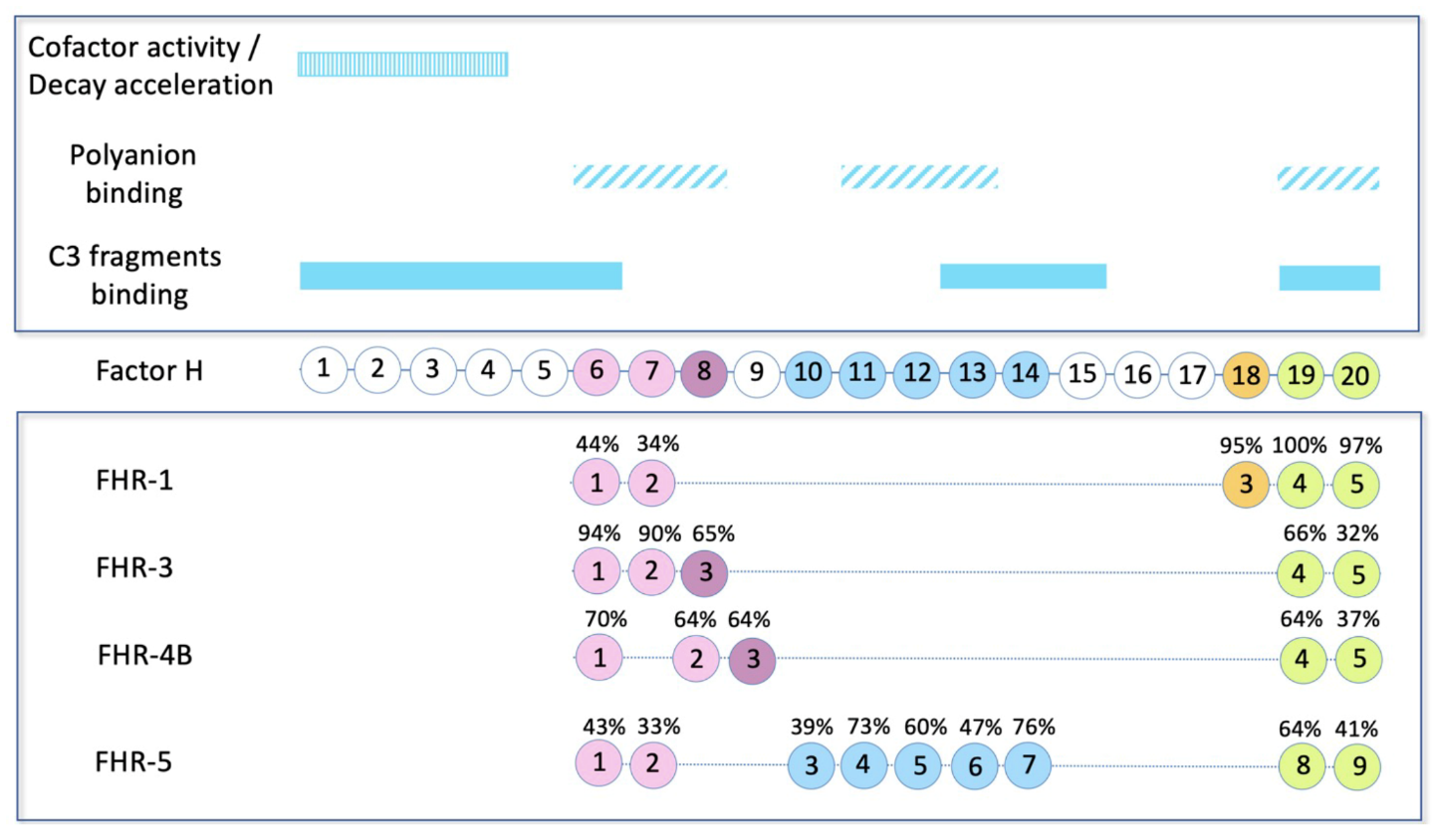


**Supplementary Figure 5**: **Schematic representation of Factor H and Factor H-related proteins.**  Factor H (FH) containing 20 complement control protein domains is shown. Top panel: FH domains necessary for its regulatory function and domains that can bind to C3 fragments and/or polyanions are indicated. Bottom panel: FH-related proteins (FHRs)-1, -3, -4B, & 5 used in this study. The percentage (%) of sequence identity to the polyanion- and C3b-binding regions of FH (indicated by respective colors) are shown.
